# Quantification of 18F Flutemetamol PET SUVr to Centiloid scale: a local clinical application

**DOI:** 10.1002/alz70856_100094

**Published:** 2025-12-25

**Authors:** Eva YW Cheung, Henry Mak, Yat Fung Shea, Patrick Ka‐Chun Chiu, Mary S M Ip

**Affiliations:** ^1^ The University of Hong Kong, Hong Kong, Hong Kong; ^2^ Queen Mary Hospital, Hong Kong, Hong Kong; ^3^ University of Hong Kong, Hong Kong, Hong Kong

## Abstract

**Background:**

Amyloid‐β (Aβ) is one of the detectable changes within the brain during pathogenesis of Alzheimer diseases (AD). The brain regional Aβ load can be assessed via PET/CT scan with 18F‐labelled radiotracers. In clinical practice, the relative standard uptake value (SUVr) of regions of interest (ROI) will be calculated based on the SUV of brain regions and compared to that of whole cerebellum. However, various radiotracers uses different cut‐off for positive scan. Centiloid scaling (CL) was developed to simplify the Aβ PET results across sites and studies, facilitates inter‐tracer comparison and Aβ treatment prescription.

**Method:**

This project composed 4 parts. 34 young healthy control (HC) and 45 AD subjects were download from the Global Alzheimer's Association Information Network (GAAIN; http://www.gaain.org). The first part is to reproduce the of PIB‐PET image analysis method, to scale its SUVr to 0‐100 CL based on Klunk et al 2015 study. The second part is to sacle the 18F‐Flutemetamol PET SUVr to the PIB‐PET SUVr, using another cohort of 24 HC and 50 AD subjects who received both 18F‐Flutemetamol PET to the PIB‐PET. The third part was using local cohort: 16 AD, 20 mild cognitive impairment (MCI) and 16 HC who received 18F‐Flutemetamol PET, to correlate with the result of second part. The final part is to obtain the SUVr from GE software CortexID and calculate the CL directly for comparison. Correlation analysis and calibration curves of each part were obtained. R‐square were used to assess the calibration results.

**Result:**

In the first and second part, correlation analysis obtained a slope of 1.0147 and 1.3029 with R2 of 0.9816 and 1 respectively, which met the expectation of calibration. For the local application, a linear correlation is obtained for SUVr obtained in image retrieved vs SUVr in cortex ID, with SUVr(cortexID)=SUVr(image)*0.9746, with R2 of 0.9968. The final CL can be calculated directly with the formula CL=124.58* SUVr(cortexID)‐121.16

**Conclusion:**

This study achieved the aim to obtained a direct conversion formula for SUVr (Cortex ID) to CL. This would help clinicians for image reporting, compare the Aβ PET results across sites and studies, and facilitate Aβ treatment prescription.

